# Interaction of Human Behavioral Factors Shapes the Transmission of Arboviruses by *Aedes* and *Culex* Mosquitoes

**DOI:** 10.3390/pathogens12121421

**Published:** 2023-12-06

**Authors:** Aubane Renard, Fernanda Pérez Lombardini, Mitsuri Pacheco Zapata, Thibaud Porphyre, Ana Bento, Gerardo Suzán, David Roiz, Benjamin Roche, Audrey Arnal

**Affiliations:** 1 Maladies Infectieuses et Vecteurs: Ecologie, Génétique, Evolution et Contrôle (MIVEGEC), Institut de Recherche Pour le Développement (IRD), Centre National de la Recherche Scientifique (CNRS), Université de Montpellier, 34394 Montpellier, France; aubanernd@gmail.com (A.R.); davidroiz@gmail.com (D.R.); benjamin.roche@ird.fr (B.R.); 2 Fauna Silvestre y Animales de Laboratorio, Departamento de Etología, Facultad de Medicina Veterinaria y Zootecnia, Universidad Nacional Autónoma de México (UNAM), Ciudad de México 04510, Mexico; fernandapl.fmvz@gmail.com (F.P.L.); mitsupacheco@gmail.com (M.P.Z.); gerardosuz@gmail.com (G.S.); 3 International Joint Laboratory IRD/UNAM ELDORADO (Ecosystem, Biological Diversity, Habitat Modifications, and Risk of Emerging Pathogens and Diseases in Mexico), Merida 97205, Mexico; 4 Laboratoire de Biométrie et Biologie Évolutive, VetAgro Sup, Campus Vétérinaire de Lyon, 69280 Marcy-l’Etoile, France; thibaud.porphyre@vetagro-sup.fr; 5 Department of Public and Ecosystem Health, College of Veterinary Medicine, Cornell University, Ithaca, NY 14850, USA; anisabelbento@gmail.com

**Keywords:** arboviruses, risk factors, human behavior

## Abstract

Arboviruses, i.e., viruses transmitted by blood-sucking arthropods, trigger significant global epidemics. Over the past 20 years, the frequency of the (re-)emergence of these pathogens, particularly those transmitted by Aedes and Culex mosquitoes, has dramatically increased. Therefore, understanding how human behavior is modulating population exposure to these viruses is of particular importance. This synthesis explores human behavioral factors driving human exposure to arboviruses, focusing on household surroundings, socio-economic status, human activities, and demographic factors. Household surroundings, such as the lack of water access, greatly influence the risk of arbovirus exposure by promoting mosquito breeding in stagnant water bodies. Socio-economic status, such as low income or low education, is correlated to an increased incidence of arboviral infections and exposure. Human activities, particularly those practiced outdoors, as well as geographical proximity to livestock rearing or crop cultivation, inadvertently provide favorable breeding environments for mosquito species, escalating the risk of virus exposure. However, the effects of demographic factors like age and gender can vary widely through space and time. While climate and environmental factors crucially impact vector development and viral replication, household surroundings, socio-economic status, human activities, and demographic factors are key drivers of arbovirus exposure. This article highlights that human behavior creates a complex interplay of factors influencing the risk of mosquito-borne virus exposure, operating at different temporal and spatial scales. To increase awareness among human populations, we must improve our understanding of these complex factors.

## 1. Introduction

In recent decades, there has been a significant (re-)emergence of arboviruses transmitted to humans or animals by blood-sucking arthropods, like mosquitoes, with global health, social, and economic consequences [1,2]. Notable examples include the Zika virus pandemics that significantly affected Latin America [3], the emergence of the West Nile virus in North America [4], the yellow fever epidemics in Central Africa and Latin America, which triggered local outbreaks in Asia through infected international travelers [5,6], the global spread of Chikungunya virus [7], epidemics of Japanese encephalitis virus in subtropical/tropical regions of Asia and Northern Australia [8], as well as the dengue virus outbreaks in the tropics, subtropics [9], and even in temperate Europe (see https://www.santepubliquefrance.fr/, accessed on 23 November 2023). These outbreaks underline the growing threat of these viruses, requiring the urgent development of integrated prevention and control strategies targeting them.

Mosquitoes from the *Aedes* and *Culex* genera are of particular concern given their potential competence as vectors for arbovirus transmission. The Chikungunya, dengue, yellow fever, and Zika viruses are mainly transmitted by *Aedes aegypti* and *Ae. albopictus* species. The global spread of these mosquito species is considerably extending the geographical distribution of potential arboviruses outbreaks [10]. Mosquito species *Culex pipiens* and *C. quinquefasciatus* are responsible for transmitting the West Nile virus, while *C. tritaeniorhyncus* mosquitoes are primary vectors of the Japanese encephalitis virus. With no effective treatments or vaccines against these mosquito-borne viruses, prevention and control strategies require vector population control, which essentially involves reducing their abundance and/or their contact with humans. While vector control methods mainly rely on the use of insecticides targeting adult mosquitoes [11], this strategy, which can effectively reduce virus transmission, cannot be considered a long-term solution without understanding its environmental and human health impacts [11]. Additionally, mosquitoes can develop resistance to these molecules/substances [12], which highlights the importance of developing effective, environmentally friendly prevention and control methods [13].

It is necessary to understand how vector ecology can impact human exposure to arboviruses [14]. For instance, the mosquito biting rate, which influences the likelihood of infecting an individual, depends on both their longevity (increases the number of bites in a lifetime) and their feeding preferences (some species, like *Aedes aegypti*, are more strictly anthropophilic than others) [15]. It is also widely documented that abiotic factors, such as climatic conditions, are pivotal in governing the spatial distribution and seasonal abundance of these vectors [16,17], as well as their competence to transmit diseases [18].

Nevertheless, the impact of human behavior that might increase the likelihood of exposure to arboviruses is considerably less studied than mosquito ecology and climate change. Human behavior—intricately woven with individual socio-economic status, household surroundings, human activities (recreational or professional) and even demographic factors—will interact with climatic factors to facilitate encounters with the vector. For instance, the presence of stagnant water bodies near dwellings, due to poor water management or sanitation systems, can create suitable breeding grounds for mosquitoes, intensifying human-vector interaction, and hence, the potential for viral transmission [19]. Similarly, working outdoors may increase the likelihood of being bitten by a mosquito, thereby elevating the risk of certain arbovirus transmission [20]. Therefore, understanding these behavioral factors, along with their interplay with the climatic factors, is critical in developing effective, targeted, and sustainable strategies for arbovirus prevention and control.

In this context, the aim of this synthesis is to identify the main exposure factors to mosquito-borne arboviruses associated with human behavior. This study compiles scientific articles associated with exposure factors potentially linked to human behavior, interacting with climate factors, thus shedding light on their crucial role in shaping interactions with vectors and their implication for disease transmission and prevention.

## 2. Materials and Methods

All scientific articles were identified and selected from PubMed, Scopus, and the ISI Web of Science (v5.13.1), which includes numerous relevant databases. To ensure thoroughness, this was completed through Google Scholar (https://scholar.google.com, accessed on 23 November 2023). This selection spanned the period from 1978 to 2020, using the search terms “Arbovirus exposure factors in the world”, and with the related keywords obtained from the Web Medical Subject Headings site (a site that allows defining related vocabulary terms, which can aid in conducting more precise and exhaustive research, https://meshb.nlm.nih.gov/search, accessed on 23 November 2023). The keywords are: “flavivirus OR dengue OR chikungunya, zika OR japanese encephalitis OR yellow fever OR west nile AND exposure factors OR risk factors OR socio-economic factors OR anthropological factors”.

For all the retrieved articles, an initial selection was made based on titles and abstracts to target manuscripts related to potential human exposure factors to arboviruses. This step excluded articles about clinical cases, therapeutic options, and laboratory-conducted molecular studies. The preselected articles were then thoroughly read. The selected studies aimed to encompass descriptive or analytical information regarding factors related to vector-borne disease exposure, especially associated with human behavior and irrespective of the study geography. Eligible articles could stem from literature reviews, comparative serological studies or publications describing local links between cases and documented human behaviors.

The last step focused on extracting relevant information from each of the selected articles. The obtained table included (1) the viruses involved, (2) the associated vector mosquito genera, (3) geographic information about the study, (4) the type of area (urban, rural), (5) exposure factors such as the nature of household surroundings (type of water storage, presence of waste, etc.), socio-economic status (income, education, etc.), human activities (work or leisure), demographic factors (sex, age, etc.), climate, and (6) more specific details about the study, for instance about the exposure factor linked to socio-economic status, it will be specified if it is related to low education, a low income, lack of access to healthcare, etc.

## 3. Results

Out of a total of 1925 screened articles, only 27 investigated human exposure factors to arboviruses transmitted by *Aedes* and *Culex* mosquitoes (resulting in a total of 113 observations). These 27 selected articles were used to conduct this synthesis. Several exposure factors, summarized by category (see Table 1), were identified: household surroundings (44.2% of the observations identified, such as the lack of water access, the presence of waste near dwellings…), socio-economic status (23.1%, such as low income, low education…), human activities (17.7%, such as outdoor activities (recreational or professional), the use of insecticide in the house…), and demographic factors (15,0%, the gender or sex effect on seroprevalence and human movement between cities). The number of observations varies depending on the viruses considered: dengue virus (37.2% of the identified observations), West Nile virus (16.8%), Chikungunya virus (16.8%), Japanese encephalitis virus (15.0%), Zika virus (11.5%), and yellow fever virus (2.7%).

As expected, the nature of household surroundings bears significantly on this exposure. The lack of water access or storage around households is frequently encountered in the literature as impacting the transmission of Chikungunya, dengue, yellow fever, and Zika viruses ([19,20,27,29,30,31,32,44], see Table 1). The presence of water points favor exposure to Chikungunya, dengue, Japanese encephalitis, and West Nile viruses [22,37,38,39,40,41]. The house building materials used could also play a potential role in exposure to Chikungunya, dengue, and Japanese encephalitis viruses [21,23,31,32,37]. Moreover, factors such as urbanization can significantly enhance the seroprevalence of the Chikungunya and dengue viruses in settings compared to rural areas [19,21,29]. However, regarding Japanese encephalitis, while studies report its occurrence in urban areas, the evidence does not support urbanization as a prominent risk factor specifically associated with increased Japanese encephalitis prevalence compared to rural settings [19]. The presence of public transportation [32], people’s movement between cities [9], and high social connections [28], also play a role in the transmission dynamics of the dengue virus. Finally, both Japanese encephalitis and dengue viruses can be affected by a high number of people in the household [19,34]. The geographical closeness of livestock rearing (examples include pig and chicken farms), rice cultivation, or pastures escalate the risk of Japanese encephalitis exposure [19,20,37]. Dengue virus is influenced by the presence of various waste materials such as tin cans, plastic waste, and discarded items [30,35]. Additionally, the absence of proper air conditioning can promote exposure to the dengue virus [33].

Socio-economic status could also have an impact on arbovirus exposure (see Table 1). Numerous studies have shown a positive association between low income or unemployment and an increased incidence of arboviral infections and exposure [9,21,23,24,29,30,33,34,36,39,43,44]. This trend is observed for all viruses except yellow fever, where socio-economic data is unavailable. Additionally, low education and literacy can affect the exposure to dengue, Chikungunya, and Japanese encephalitis viruses [21,23,24,31,36,37]. Finally, the lack of access to healthcare can result in delays in diagnosing and treating Zika virus infections, potentially worsening the spread of the disease [44].

Furthermore, human activities also contribute to the propagation of arboviruses. Outdoor activities, whether recreational or professional, such as grazing, fishing, hunting, or gardening, substantially increase the risk of viral exposure [20,22,38,39,41,42,43]. This is observed for all viruses, except for the Zika virus. The use of insecticides is a behavior that limits the exposure to West Nile and Chikungunya viruses [24,41,43]. The probability of contact with dead birds can impact the likelihood of exposure to the West Nile virus [39]. Lastly, the only human behavior factor impacting exposure to the Zika virus is related to unprotected sexual practices [19].

The exposure to arboviruses varies based on demographic factors, and depends on the specific virus, geographic location, and years of study. Age is a factor that impacts the exposure to all considered viruses [19,22,25,26,37,38,39]. Studies indicate that the seroprevalence rates for Japanese encephalitis (in India and Vietnam, [19,25,37], Chikungunya, and Zika viruses (both in Kenya, [22,38] are higher in young individuals, under 20 years old. The West Nile (in the United States and Kenya, [26,38,39] and yellow fever viruses (in Kenya, [26]) show seroprevalence rates that increase throughout life. However, the seroprevalence rates for the dengue virus can vary across different age groups, depending on the country (in Vietnam and Kenya, [22,25,26]) and even within the same region of a country but across different years [22,26]. Gender is also a factor observed for all viruses [21,22,37,38] except yellow fever, whose exposure does not appear to be gender-specific. Japanese encephalitis, West Nile, and Zika viruses appear to affect men more [37,38], while dengue virus tends to preferentially affect females [22]. However, the affected genders for Chikungunya differ according to studies. In Mayotte, this virus appears to affect men more often [21], while in Kenya it preferentially infects women [22].

## 4. Discussion

Our synthesis, which screened a large number of articles, ultimately resulted in the inclusion of 27 papers that investigated the importance of human behavior in exposure to arboviruses transmitted by *Aedes* and *Culex* mosquitoes. The studies that emerged from our selection were only focused on transmission by the *Aedes* and *Culex* genera; no studies specifically addressed *Anopheles*. This is likely due to the significant role of *Aedes* and *Culex* in the transmission of arboviral diseases. While *Anopheles* mosquitoes can also harbor viruses, this genus has been poorly studied on this topic [45]. The results of our analysis highlight the wide range and intricate role of household surroundings, socio-economic status, human activities, and demographic factors in the transmission and exposure to arboviruses.

The study highlights how household surroundings significantly influence arbovirus exposure. One important factor that emerges from this study is the lack of water access or storage, leading to the presence of stagnant water bodies near residences, creating ideal breeding grounds for *Aedes* mosquitoes and affecting the transmission of the viruses they carry (Chikungunya, dengue, yellow fever and Zika viruses; [19,20,27,29,30,31,32,44]; see Table 1). While the reduction of larval breeding sites can be (and is already, [46,47,48]) used as an effective method to mitigate the risk of arbovirus transmission, access to water remains challenging in certain countries (see https://www.who.int/news/item/18-06-2019-1-in-3-people-globally-do-not-have-access-to-safe-drinking-water-unicef-who, accessed on 23 November 2023). This forces residents to store varying amounts of water near their homes, which can act as potential *Aedes aegypti*, *Ae. albopictus* and *Cx. quinquefasciatus* larval breeding sites [49]. In contrast, larger bodies of water provide conducive environments for the development of other *Aedes* and *Culex* mosquitoes responsible for the transmission of Chikungunya, dengue, Japanese encephalitis, and West Nile viruses [22,32,37,38,39,40,41]. The house building materials used could also play a potential role in vector exposure [21,23,31,32,37]. For example, houses constructed from cob (e.g., [37]) or with a tin roof (e.g., [32]) are more susceptible to the infiltration of both *Aedes aegypti* and *Culex quinquefasciatus* mosquitoes (although *Aedes* species are much more anthropophilic than *Culex* mosquitoes) compared to those built from stone or other more solid materials (see Table 1). Moreover, population density, closely associated with urbanization, emerges as a crucial factor influencing the exposure to arboviruses. Studies have demonstrated that urban areas, with their higher population density compared to rural communities, exhibit a substantial increase in the seroprevalence of Chikungunya and dengue viruses [19,21,29]. Additionally, the existence of public transportation systems [32], people’s movement between cities [9], and stronger social connections [28], predominantly found in urban areas, contribute to the transmission dynamics of the dengue virus. Lastly, both dengue and Japanese encephalitis viruses can be influenced by larger household sizes, with a higher number of individuals potentially increasing the risk of infection [19,34]. Exposure factors can be virus-specific related to the close proximity of animal farms that can significantly raise the chances of human-vector interactions, thereby amplifying the risk of exposure to Japanese encephalitis [19,20,37]. This can be explained by the fact that, in contrast to highly anthropophilic *Aedes* mosquitoes [15], *Culex* mosquitoes, which are vectors of Japanese encephalitis, have a wider range of potential hosts and frequently feed on birds, although they also bite humans and other mammals [50]. The presence of domestic animals (especially chickens, but not only) in the vicinity enhances their presence and increases the likelihood of biting humans [19,20,37]. The presence of rice cultivation near residences also promotes Japanese encephalitis exposure by providing a larval habitat for *Culex* mosquitoes [19,20,37]. The lack of efficient waste management is a factor among household surroundings. The presence of tin cans, plastic waste, and discarded items can serve as favorable breeding sites for *Aedes* mosquito larvae [30,35] and exposure to dengue [30,35]. However, although not explicitly demonstrated in this study for the other *Aedes*-transmitted viruses, it can be assumed that they may also be impacted by the presence of waste. Finally, the absence of air conditioning, characterized by open windows and doors in houses and shops, can facilitate anthropophilic *Aedes* mosquito entry into homes, and can promote exposure to the dengue virus [33]. The use of more insulating materials or physical barriers (such as mosquito nets) to prevent mosquito entry into houses can be a means of combating arbovirus exposure [51], but they are directly connected to socio-economic factors, as individuals with lower incomes often live in precarious or unsanitary housing conditions.

According to our study, socio-economic factors, are closely intertwined with household surroundings and human activity factors and can impact exposure to all of the arboviruses selected in this study (except yellow fever, where socio-economic data is unavailable) in several ways. First, unemployed individuals or with low incomes, are more likely to live in precarious and unsanitary conditions [32,37] with the presence of waste [30,35] or a lack of access to water [29,34,44] thereby creating environments conducive to the proliferation of mosquito vectors of arboviruses. Additionally, low incomes can limit individuals’ access to personal protective measures such as purchasing mosquito repellents or bed nets, further exposing them to mosquito bites and increasing their risk of arbovirus infection [24,33,41,43]. Lastly, this factor can also restrict access to healthcare and resources needed to prevent and treat arbovirus infections, as observed in the case of the Zika virus [44]. Low incomes are often associated with low levels of education or literacy, which also contribute to viral transmission [21,23,24,31,36,37]. A lack of knowledge about mosquito-borne diseases can lead to risky behaviors and poor prevention practices, such as the importance of eliminating mosquito breeding sites around homes.

The data also demonstrate the significance of human activities in the transmission and exposure to arboviruses, with outdoor activities being one of the most important factors facilitating human-mosquito contact [20,22,38,39,41,42,43]. This includes all recreational or professional activities such as gardening, fishing, hunting, regardless of the time of day (see Table 1). Indeed, *Aedes* and *Culex* mosquitoes have different biting behaviors. *Aedes* mosquitoes are primarily active during the day and tend to bite multiple times to feed [52]. In contrast, *Culex* mosquitoes are predominantly active at night and typically have a single blood meal per night [53]. Despite this difference in behavior, outdoor activities have been observed to be associated with exposure to all viruses transmitted by both *Aedes* and *Culex* mosquitoes [20,22,38,39,41,42,43], except for the Zika virus. This study also revealed more specific factors for each virus. The use of insecticides is a behavior that limits the presence of mosquitoes in homes and appears to have an impact on the transmission of West Nile and Chikungunya viruses [24,41,43]. However, this factor is directly linked to the level of awareness among populations (knowing the link between mosquito presence and the diseases they transmit) as well as socio-economic factors (as insecticides have a significant cost). Exposure to the Zika virus can depend on unprotected sexual practices [19]. Indeed, even though the Zika virus is primarily transmitted to humans through the bite of an infected mosquito, the virus can be present in semen, vaginal fluids, and blood and can be sexually transmitted [54,55,56]. Finally, a study has shown a positive correlation between the probability of contact with dead birds and the likelihood of transmission to the West Nile virus [39] whose transmission cycle is closely linked to avian species [57].

Age and gender can have an impact on arbovirus exposure due to biological, behavioral, and social differences among age groups and genders. Regarding age, some studies have shown higher seroprevalence rates among younger individuals for arboviruses such as Chikungunya, Japanese encephalitis, and Zika viruses [19,22,25,37,38]. This may be attributed to factors such as acquired immunity or more frequent exposure to infected mosquitoes among the younger population. Gender can also play a role in arbovirus exposure. For example, some studies have shown that men may be more exposed to Japanese encephalitis, West Nile, and Zika viruses [37,38], while women may be more exposed to the dengue virus [22]. These differences can be attributed to specific gender-related behaviors. For example, men tend to work more in agricultural areas or livestock farming, which exposes them to a wide range of arboviruses [37,38]. Conversely, women may be more involved in domestic activities and spend more time indoors, where they are more likely to be exposed to *Aedes* mosquito bites [15]. However, it is important to note that in this study, the specific impact of age and gender may vary depending on the virus studied, geographic location, and even the year of study [21,22,25,26]. The exposure to Chikungunya varies by gender according to research. For instance, in Mayotte, men seem to be more affected by this virus [21], whereas in Kenya, it predominantly infects women [22] Also, regarding the dengue virus, the seroprevalence rates can vary across different age groups, depending on the country (Vietnam and Kenya, [22,25]), and even within the same region of a country (Busia in Kenya, [22,26]) but across different years. This variability can be due to specific local factors, such as environmental, socio-economic, and demographic factors unique to each region or study period. Therefore, it is essential to consider these variations to better understand the impact of age and gender on arbovirus exposure and to tailor prevention and control strategies based on the specific characteristics of each region or population studied.

Previous studies have already focused on exposure factors to arboviruses. This is the case with the study by Esser et al. (2019) whose objective was to provide baseline information on suitable ecological conditions under which outbreaks of six arboviruses of high public health priority can occur [58]. However, our study provides several new perspectives that contribute to the understanding of human exposure factors to arboviruses. Indeed, while this previous study focused on ecological variables that may promote virus circulation, including variables related to climate (e.g., precipitation, humidity, temperature), habitat (e.g., habitat fragmentation, normalized difference vegetation index), or vector and host ecology (e.g., population density, migration), our study brings a new perspective by highlighting the importance of human behavior in exposure to arboviral diseases. Another study [59], addressed the impact of socio-economic factors on arbovirus transmission. The objective of this previous systematic review and meta-analysis study was to assess the relationship between markers of socio-economic position (SEP) and infection due to arboviruses with mosquito vectors [59]. This was achieved through the analysis of observational studies published between 1980 and 2020 that measured the association of SEP markers with arbovirus infection, with the goal of informing targeted public health interventions. However, beyond the heterogeneity of measures used to capture the range of socio-economic factors analyzed in these studies, which can make it difficult to delineate associations of interest, it is important to underline that this study only addresses the impact of socio-economic factors. However, our study shows the importance of other factors such as household surroundings, human activities, etc. To reinforce the added value of our study, it’s crucial to emphasize that our analysis stands out by comprehensively considering several factors that were not considered previously, such as household environments and human activities. In contrast to previous studies focusing on specific ecological or socioeconomic variables, our approach offers a more holistic view of arbovirus exposure. While these local variables are challenging to compare with broader-scale factors like climatic conditions or socioeconomic indicators, they improve our understanding of arboviral exposure.

In order to gain a more comprehensive understanding of the human exposure factors to arboviruses, there is a pressing need for the development of truly interdisciplinary studies considering explicitly human behavior. These studies should strive to holistically explore a spectrum of contributing factors, assessing the relative weight of each in shaping exposure risks. By so doing, these efforts can form the basis for well-informed, effective strategies to mitigate the threat of arbovirus transmission. Furthermore, the influence of specific virus species and geographical locations on exposure risk should be taken into account, as we observed that the risk of exposure can vary significantly based on these variables.

However, our insights should be contextualized within the constraints of our study. Given that our research did not constitute a systematic review, it was still able to identify some important trends in the literature. To mitigate bias in our article selection process, we employed a diverse range of sources and conducted a rigorous double-check of the scientific and methodological quality of the chosen articles. A comprehensive systematic review on this topic was not feasible due to a marked bias in the available literature, with studies typically focusing on a particular factor of interest. This directly affects the perceived importance of each factor, being largely influenced by the number of studies conducted on each. Our study, in this regard, serves more as an illustration of these factors and their intersectionality in arbovirus transmission, but does not aim to provide any quantification of their importance. This limitation was further compounded by the limited number of articles that have successfully demonstrated and quantified the factors contributing to arbovirus exposure. In addition to this limitation regarding the number of articles, there is also an imbalance in the number of observations obtained for each studied virus. Indeed, 37.2% of the observations focus on the dengue virus, while the yellow fever and the Zika viruses account for only 14.2% of the total observations.

This difference in the number of observations can be attributed to the epidemiology of arboviruses. Endemic viruses, such as dengue or Chikungunya, are constantly present in certain populations/regions, or regular epidemic outbreaks can occur [60,61], which may facilitate the implementation of studies on the impact of human behavior on the transmission of these viruses. Conversely, more sporadic zoonotic viruses, like Japanese encephalitis and West Nile viruses [62,63,64], can be a limiting factor in setting up studies on human populations. The few studies observed on the Zika virus may be explained by the fact that this virus was responsible for a pandemic between 2015 and 2016 [65,66], but which had consequences only over a short period of time, despite the severity of the consequences for fetuses (notably microcephaly). Finally, the few observations for the yellow fever virus, may be attributed to a relative effectiveness of vaccination programs [67], even if the disease remains a threat in some tropical regions. This imbalance among the studied viruses, coupled with the scarcity of comprehensive data, underscores the need for more exhaustive research to fully understand the multifaceted interplay of factors that influence arbovirus transmission.

Knowledge, attitudes, and practices (KAP) studies provide valuable insight into the ways individuals and communities understand, perceive, and respond to health-related issues, such as arboviral diseases [68,69]. By capturing what people know, how they feel, and what they do about a specific health concern, KAP studies can identify gaps in knowledge, misconceptions, harmful practices, and behavioral patterns that may contribute to the risk of disease transmission. In the context of arboviral diseases, KAP studies can help unravel complex human behaviors, such as outdoor activity patterns or water storage practices, which may inadvertently promote the breeding of *Aedes* and *Culex* mosquitoes. Moreover, these studies can highlight cultural and socio-economic factors that may hinder or enable the effective implementation of preventive measures. Understanding these factors is critical to tailoring health education and intervention programs that are culturally acceptable, economically feasible, and likely to produce sustainable behavior change.

In tandem with KAP studies, digital epidemiology or “digital health” is emerging as an important tool in understanding arboviral exposure factors [70]. Leveraging the power of digital data, sourced from online search queries, social media posts, mobile health apps, and other digital platforms, digital epidemiology provides real-time, large-scale insights into population health trends and behaviors [71]. When applied to arboviral diseases, it can track disease outbreaks, monitor public responses, and predict future disease spread based on environmental and climatic factors. Importantly, digital epidemiology can also capture the impact of socio-behavioral factors on disease spread. For instance, by analyzing social media discussions or search patterns, it can gauge public awareness, perceptions, and misconceptions about arboviruses. This wealth of digital information, when coupled with traditional epidemiological data and findings from KAP studies, can guide the design of timely, targeted, and effective public health responses to arboviral diseases. However, these methods have a bias related to access to digital tools (phones, computers, etc.) that enable the recording of this data, especially for the poorest or most remote populations. This bias must be taken into account when interpreting the results of digital epidemiology studies.

In conclusion, the complexity of arbovirus exposure needs a comprehensive, multifaceted approach to research. This involves not only understanding the broad range of factors that influence exposure, but also harnessing diverse research methodologies, from KAP studies to digital epidemiology. These efforts will underpin effective strategies for mitigating the threat of arbovirus transmission through developing a societal approach for outbreak prevention.

## Figures and Tables

**Table 1:** Summary table of bibliographic data on the impact of human behavior on exposure to arboviruses. NA (Not Available).

Virus	Vector	Continent	Country	State/Province/Region	Area	Exposure Factors	Study Details	References
Chikungunya	Aedes	Africa	France	Mayotte	NA	Demographic factors	Effect of gender (Higher seroprevalence in men than in women)	[21]
Chikungunya	Aedes	Africa	Kenya	Busia	Rural	Demographic factors	Effect of gender (Higher seroprevalence in women than in men)	[22]
Chikungunya	Aedes	Africa	Kenya	Busia	Rural	Demographic factors	Effect of age (high seroprevalence among children than adults)	[22]
Chikungunya	Aedes	Africa	Kenya	Busia	Rural	Household surroundings	Presence of water points	[22]
Chikungunya	Aedes	Africa	France	Mayotte	Urban	Household surroundings	Housing conditions (Materials used, unsanitary conditions…)	[23]
Chikungunya	Aedes	Africa	France	Mayotte	NA	Household surroundings	Effect of urbanization (higher seroprevalence in cities than in rural communities)	[21]
Chikungunya	Aedes	Africa	France	Mayotte	NA	Household surroundings	Housing conditions (Materials used, unsanitary conditions…)	[21]
Chikungunya	Aedes	Africa	Kenya	Busia	Rural	Human Activities	Outdoor activities (fishing)	[22]
Chikungunya	Aedes	Africa	Kenya	Busia	Rural	Human Activities	Outdoor activities	[22]
Chikungunya	Aedes	Africa	France	Reunion	NA	Human Activities	Lack of insecticide use	[24]
Chikungunya	Aedes	Africa	Kenya	Busia	Rural	Human Activities	Outdoor activities (grazing)	[22]
Chikungunya	Aedes	Africa	Kenya	Busia	Rural	Human Activities	Outdoor activities (hunting)	[22]
Chikungunya	Aedes	Africa	France	Mayotte	Urban	Socio-Economic Status	Low income	[23]
Chikungunya	Aedes	Africa	France	Mayotte	Urban	Socio-Economic Status	Low education	[23]
Chikungunya	Aedes	Africa	France	Reunion	NA	Socio-Economic Status	Low education	[24]
Chikungunya	Aedes	Africa	France	Reunion	NA	Socio-Economic Status	Low income	[24]
Chikungunya	Aedes	Africa	France	Mayotte	NA	Socio-Economic Status	Presence of people living in poverty	[21]
Chikungunya	Aedes	Africa	France	Mayotte	NA	Socio-Economic Status	Low income	[21]
Chikungunya	Aedes	Africa	France	Mayotte	NA	Socio-Economic Status	Low education	[21]
Dengue	Aedes	Asia	Vietnam	Dong Thap	Rural/Urban	Demographic factors	Effect of age (high seroprevalence in the 0–15 age group)	[25]
Dengue	Aedes	Africa	Kenya	Busia	Rural	Demographic factors	Effect of gender (Higher seroprevalence in women than in men)	[22]
Dengue	Aedes	Africa	Kenya	Busia	Rural	Demographic factors	Effect of age (high seroprevalence among children than adults)	[22]
Dengue	Aedes	Africa	Kenya	Busia	Rural	Demographic factors	Effect of age (increase in seroprevalence with age)	[26]
Dengue	Aedes	America	Mexico	Sonora	Urban	Household surroundings	Lack of access to water	[27]
Dengue	Aedes	Asia	Vietnam	Dong Thap	Rural/Urban	Household surroundings	Effect of urbanization (higher seroprevalence in cities than in rural communities)	[25]
Dengue	Aedes	Africa	Kenya	Busia	Rural	Household surroundings	Presence of water points	[22]
Dengue	Aedes	Africa	Kenya	Busia	Rural	Human Activities	Outdoor activities (fishing)	[22]
Dengue	Aedes	Africa	Kenya	Busia	Rural	Human Activities	Outdoor activities	[22]
Dengue	Aedes	Africa	Kenya	Busia	Rural	Human Activities	Outdoor activities (grazing)	[22]
Dengue	Aedes	Africa	Kenya	Busia	Rural	Human Activities	Outdoor activities (hunting)	[22]
Dengue	Aedes	America	Peru	NA	Urban	Household surroundings	High social connections	[28]
Dengue	Aedes	Asia	Saudi Arabia	Jeddah	Urban	Household surroundings	High construction density	[29]
Dengue	Aedes	America	Mexico	Tamaulipas	Urban	Household surroundings	Water storage practices	[30]
Dengue	Aedes	America	Mexico	Tamaulipas	Urban	Household surroundings	Presence of discarded	[30]
Dengue	Aedes	America	Mexico	Tamaulipas	Urban	Household surroundings	Lack of street water drainage	[30]
Dengue	Aedes	America	Equator	El oro	Urban	Household surroundings	Water storage practices	[31]
Dengue	Aedes	Asia	Thailand	NA	Urban	Household surroundings	Housing conditions (Materials used, unsanitary conditions…)	[32]
Dengue	Aedes	Asia	Saudi Arabia	Jeddah	Urban	Household surroundings	High population density	[29]
Dengue	Aedes	Asia	Saudi Arabia	Jeddah	Urban	Household surroundings	Lack of access to water	[29]
Dengue	Aedes	Worldwide	NA	NA	Urban	Household surroundings	Human movement between cities	[9]
Dengue	Aedes	Asia	Thailand	NA	Urban	Household surroundings	Presence of public transportation	[32]
Dengue	Aedes	Asia	Thailand	NA	Urban	Household surroundings	Presence of public water wells	[32]
Dengue	Aedes	Asia	Vietnam	NA	Urban	Household surroundings	High population density	[19]
Dengue	Aedes	Asia	Vietnam	NA	Urban	Household surroundings	Water storage practices	[19]
Dengue	Aedes	America	Mexico	Tamaulipas	Urban	Household surroundings	Lack of air conditioning (the windows and doors of shops and houses remain open)	[33]
Dengue	Aedes	America	Equator	El oro	Urban	Household surroundings	Lack of access to water	[31]
Dengue	Aedes	America	Equator	El oro	Urban	Household surroundings	Housing conditions (Materials used, unsanitary conditions…)	[31]
Dengue	Aedes	Oceania	France	New Caledonia	NA	Household surroundings	High number of people in the household	[34]
Dengue	Aedes	America	Salvador	NA	Urban	Household surroundings	Presence of discarded car tires	[35]
Dengue	Aedes	America	Salvador	NA	Urban	Household surroundings	Presence of tin cans	[35]
Dengue	Aedes	America	Salvador	NA	Urban	Household surroundings	Presence of plastic waste	[35]
Dengue	Aedes	Asia	Thailand	NA	Urban	Household surroundings	Water storage practices	[32]
Dengue	Aedes	Asia	Saudi Arabia	Jeddah	Urban	Socio-Economic Status	Low income	[29]
Dengue	Aedes	America	Mexico	Tamaulipas	Urban	Socio-Economic Status	Low income	[30]
Dengue	Aedes	Worldwide	NA	NA	Urban	Socio-Economic Status	Low income	[9]
Dengue	Aedes	America	Mexico	Tamaulipas	Urban	Socio-Economic Status	Low income	[33]
Dengue	Aedes	America	Brazil	Goias	Urban	Socio-Economic Status	Low income	[36]
Dengue	Aedes	America	Brazil	Goias	Urban	Socio-Economic Status	Low education	[36]
Dengue	Aedes	America	Equator	El oro	Urban	Socio-Economic Status	Low education	[31]
Dengue	Aedes	Oceania	France	New Caledonia	NA	Socio-Economic Status	Low income	[34]
Dengue	Aedes	Oceania	France	New Caledonia	NA	Socio-Economic Status	Unemployment rate	[34]
Japanese encephalitis	Culex	Asia	Vietnam	NA	Rural	Demographic factors	Effect of age (high seroprevalence in the 0–14 age group)	[19]
Japanese encephalitis	Culex	Asia	India	West Bengal	Rural	Demographic factors	Effect of gender (Higher seroprevalence in men than in women)	[37]
Japanese encephalitis	Culex	Asia	India	West Bengal	Rural	Demographic factors	Effect of age (high seroprevalence in the 0–20 age group)	[37]
Japanese encephalitis	Culex	Asia	Vietnam	Dong Thap	Rural/Urban	Demographic factors	Effect of age (high seroprevalence in the 0–15 age group)	[25]
Japanese encephalitis	Culex	Asia	Vietnam	NA	Rural	Household surroundings	Presence of pig farms	[19]
Japanese encephalitis	Culex	Asia	Vietnam	NA	Rural	Household surroundings	High number of people in the household	[19]
Japanese encephalitis	Culex	Asia	NA	NA	Rural	Household surroundings	Presence of pig farms	[20]
Japanese encephalitis	Culex	Asia	NA	NA	Rural	Household surroundings	Presence of rice cultivation	[20]
Japanese encephalitis	Culex	Asia	Vietnam	NA	Rural	Household surroundings	Presence of rice cultivation	[19]
Japanese encephalitis	Culex	Asia	India	West Bengal	Rural	Household surroundings	Presence of rice cultivation	[37]
Japanese encephalitis	Culex	Asia	India	West Bengal	Rural	Household surroundings	Presence of pig farms	[37]
Japanese encephalitis	Culex	Asia	India	West Bengal	Rural	Household surroundings	Presence of water points	[37]
Japanese encephalitis	Culex	Asia	India	West Bengal	Rural	Household surroundings	Presence of chicken farms	[37]
Japanese encephalitis	Culex	Asia	India	West Bengal	Rural	Household surroundings	Presence of water points	[37]
Japanese encephalitis	Culex	Asia	India	West Bengal	Rural	Household surroundings	Housing conditions (Materials used, unsanitary conditions…)	[37]
Japanese encephalitis	Culex	Asia	India	West Bengal	Rural	Socio-Economic Status	Literacy rate	[37]
Japanese encephalitis	Culex	Asia	India	West Bengal	Rural	Socio-Economic Status	Low education	[37]
West Nile	Culex	Africa	Kenya	West Pokot	Rural	Demographic factors	Effect of gender (Higher seroprevalence in men than in women)	[38]
West Nile	Culex	Africa	Kenya	West Pokot	Rural	Demographic factors	Effect of age (high prevalence among those over 50 years old)	[38]
West Nile	Culex	Africa	Kenya	Busia	Rural	Demographic factors	Effect of age (increase in seroprevalence with age)	[26]
West Nile	Culex	America	United States	Illinois	Urban	Demographic factors	Effect of age (high prevalence among those over 60 years old)	[39]
West Nile	Culex	America	United States	Texas	Rural	Household surroundings	Presence of water points	[40]
West Nile	Culex	Africa	Kenya	West Pokot	Rural	Household surroundings	Presence of water points	[38]
West Nile	Culex	Africa	Nigeria	Kwara	Urban	Household surroundings	Presence of water points	[41]
West Nile	Culex	America	United States	Illinois	Urban	Household surroundings	Presence of water points	[39]
West Nile	Culex	Asia	Iran	NA	NA	Human Activities	Outdoor activities (hunting)	[42]
West Nile	Culex	America	United States	Colorado	Urban	Human Activities	Outdoor activities	[43]
West Nile	Culex	Africa	Kenya	West Pokot	Rural	Human Activities	Outdoor activities (grazing)	[38]
West Nile	Culex	Africa	Nigeria	Kwara	Urban	Human Activities	Lack of insecticide use	[41]
West Nile	Culex	Africa	Nigeria	Kwara	Urban	Human Activities	Outdoor activities	[41]
West Nile	Culex	America	United States	Colorado	Urban	Human Activities	Lack of insecticide use	[43]
West Nile	Culex	America	United States	Illinois	Urban	Human Activities	Probability of contact with dead birds	[39]
West Nile	Culex	America	United States	Illinois	Urban	Human Activities	Outdoor activities (gardening)	[39]
West Nile	Culex	Africa	Kenya	West Pokot	Rural	Human Activities	Outdoor activities (fishing)	[38]
West Nile	Culex	America	United States	Colorado	Urban	Socio-Economic Status	Low income	[43]
West Nile	Culex	America	United States	Illinois	Urban	Socio-Economic Status	Low income	[39]
Yellow fever	Aedes	Africa	Kenya	Busia	Rural	Demographic factors	Effect of age (increase in seroprevalence with age)	[26]
Yellow fever	Aedes	Africa	NA	NA	Rural	Household surroundings	Lack of access to water	[20]
Yellow fever	Aedes	America	NA	NA	Rural/Urban	Human Activities	Outdoor activities (work)	[20]
Zika	Aedes	Africa	Kenya	West Pokot	Rural	Demographic factors	Effect of gender (Higher seroprevalence in men than in women)	[38]
Zika	Aedes	Africa	Kenya	West Pokot	Rural	Demographic factors	Effect of age (high seroprevalence in the 13–19 age group)	[38]
Zika	Aedes	Asia	Vietnam	NA	Urban	Household surroundings	Water storage practices	[19]
Zika	Aedes	America	Brazil	NA	NA	Household surroundings	Lack of access to water	[44]
Zika	Aedes	America	Colombia	NA	NA	Household surroundings	Lack of access to water	[44]
Zika	Aedes	America	Suriname	NA	NA	Household surroundings	Lack of access to water	[44]
Zika	Aedes	Asia	Vietnam	NA	Urban	Human Activities	Unsafe sexual practices	[19]
Zika	Aedes	America	Brazil	NA	NA	Socio-Economic Status	Low income	[44]
Zika	Aedes	America	Brazil	NA	NA	Socio-Economic Status	Lack of access to healthcare	[44]
Zika	Aedes	America	Colombia	NA	NA	Socio-Economic Status	Low income	[44]
Zika	Aedes	America	Colombia	NA	NA	Socio-Economic Status	Lack of access to healthcare	[44]
Zika	Aedes	America	Suriname	NA	NA	Socio-Economic Status	Low income	[44]
Zika	Aedes	America	Suriname	NA	NA	Socio-Economic Status	Lack of access to healthcare	[44]

## Data Availability

The entirety of the data used in this article is presented in the table.
